# Identification of microRNA–mRNA–TF regulatory networks in periodontitis by bioinformatics analysis

**DOI:** 10.1186/s12903-022-02150-0

**Published:** 2022-04-09

**Authors:** Xiaoli Gao, Dong Zhao, Jing Han, Zheng Zhang, Zuomin Wang

**Affiliations:** 1 Department of Stomatology, Beijing Chaoyang Hospital, Capital Medical University, 8 Gongti Nan Lu, Chaoyang District, Beijing, 100020 China; 2 Department of Periodontology, Tianjin Stomatological Hospital, School of Medicine, Nankai University, Tianjin, China; 3 Tianjin Key Laboratory of Oral and Maxillofacial Function Reconstruction, 75 Dagu Bei Lu, Heping District, Tianjin, 300041 China

**Keywords:** Periodontitis, miRNA–mRNA–TF, Regulatory networks, Bioinformatics

## Abstract

**Background:**

Periodontitis is a complex infectious disease with various causes and contributing factors. The aim of this study was to identify key genes, microRNAs (miRNAs) and transcription factors (TFs) and construct a miRNA–mRNA–TF regulatory networks to investigate the underlying molecular mechanism in periodontitis.

**Methods:**

The GSE54710 miRNA microarray dataset and the gene expression microarray dataset GSE16134 were downloaded from the Gene Expression Omnibus database. The differentially expressed miRNAs (DEMis) and mRNAs (DEMs) were screened using the “limma” package in R. The intersection of the target genes of candidate DEMis and DEMs were considered significant DEMs in the regulatory network. Next, Gene Ontology (GO) and Kyoto Encyclopedia of Genes and Genomes (KEGG) pathway enrichment analyses were conducted. Subsequently, DEMs were uploaded to the STRING database, a protein–protein interaction (PPI) network was established, and the cytoHubba and MCODE plugins were used to screen out key hub mRNAs and significant modules. Ultimately, to investigate the regulatory network underlying periodontitis, a global triple network including miRNAs, mRNAs, and TFs was constructed using Cytoscape software.

**Results:**

8 DEMis and 121 DEMs were found between the periodontal and control groups. GO analysis showed that mRNAs were most significantly enriched in positive regulation of the cell cycle, and KEGG pathway analysis showed that mRNAs in the regulatory network were mainly involved in the IL-17 signalling pathway. A PPI network was constructed including 81 nodes and 414 edges. Furthermore, 12 hub genes ranked by the top 10% genes with high degree connectivity and five TFs, including SRF, CNOT4, SIX6, SRRM3, NELFA, and ONECUT3, were identified and might play crucial roles in the molecular pathogenesis of periodontitis. Additionally, a miRNA–mRNA–TF coregulatory network was established.

**Conclusion:**

In this study, we performed an integrated analysis based on public databases to identify specific TFs, miRNAs, and mRNAs that may play a pivotal role in periodontitis. On this basis, a TF–miRNA–mRNA network was established to provide a comprehensive perspective of the regulatory mechanism networks of periodontitis.

**Supplementary Information:**

The online version contains supplementary material available at 10.1186/s12903-022-02150-0.

## Background

Periodontitis is a complex immune-inflammatory condition characterized by the disruption of the periodontal ligament and subsequent formation of periodontal pockets and by alveolar bone loss, often resulting in tooth loss [1]. The amount of tissue destruction is generally commensurate with dental plaque levels, host defence, and related risk factors [2, 3]. Periodontitis contributes significantly to the overall oral disease burden, with its severe form representing the sixth most prevalent condition, estimated to affect 7–11% of the global adult population [3, 4]. The prevalence of periodontitis increases gradually with age [3], particularly in adults over 50 years of age [5]. Periodontitis is also associated with systemic conditions such as neoplastic disorders, obesity, and diabetes [1]. The etiology of periodontitis is multifactorial. Subgingival dental biofilm elicits a host inflammatory and immune response, ultimately leading to irreversible destruction of the periodontium in a susceptible host [6]; however, the precise molecular pathogenesis of periodontitis has not been completely identified.

In recent years, microRNAs (miRNAs) have gained increased attention from researchers for periodontal disease studies [7]. MiRNAs are endogenous, noncoding, small RNAs containing approximately 22 nucleotides [8]. MiRNAs regulate target genes at the transcriptional or translational level based on the sequence complementarity between miRNAs and the 3′-untranslated region of target genes [9]. An independent study found that miR-146a is highly expressed in the serum of patients with chronic periodontitis and that its expression is directly proportional to disease severity [10]. Zhou et al. suggested that miR-21 downregulates Porphyromonas gingivalis lipopolysaccharide (LPS)-induced inflammation and that miR-21 plays a protective role in periodontitis progression [11]. Akkouch et al. suggested that local treatment with miR-200c was effective for alveolar bone resorption in a rat model of periodontitis [12]. MiRNAs mediate the progression of periodontal disease in a variety of ways, (i) through their role in periodontal inflammation and the dysregulation of homeostasis, (ii) as regulatory targets of lncRNAs, (iii) by contributing to periodontal disease susceptibility through miRNA polymorphisms, and (iv) as periodontal microflora modulators via viral miRNAs [7].

Previous studies have examined the expression profile of periodontitis to identify the differentially expressed miRNAs (DEMis) and mRNAs (DEMs). Due to the limitations of the comparative analysis in independent studies, there are still problems with the interaction between DEMis and DEMs involved in periodontitis. The aim of this study was to identify the key genes, miRNAs and TFs and construct miRNA–mRNA–TF regulatory networks to investigate the underlying molecular mechanism in periodontitis.

## Methods

### Data download

We used “periodontitis” as the keyword for our search in the National Center for Biotechnology Information (NCBI) Gene Expression Omnibus (GEO) database (https://www.ncbi.nlm.nih.gov/geo/). We obtained one miRNA microarray dataset (GSE54710) containing 198 gingival papillae (158 “diseased” and 40 “healthy”) from 86 patients with periodontitis (the classification of periodontitis is unknown). Furthermore, we obtained one mRNA microarray dataset (GSE16134) containing 310 gingival papillae from 120 subjects with moderate to severe periodontitis [65 (54.2%) with chronic and 55 with aggressive periodontitis]. The criteria for selection the samples were: “Diseased” sites showed BoP, had interproximal PD ≥ 4 mm, and concomitant AL ≥ 3 mm; “Healthy” sites showed no BoP, had PD ≤ 4 mm and AL ≤ 2 mm. The former dataset was based on GPL-15159, and the latter was based on GPL570-55,599. All RNA information for the selected samples was downloaded for further analysis.

### Data pre-processing and screening strategy

We used the R (4.0.4) software to process the original matrix of GSE54710, GSE16134. The probe IDs were converted to gene symbols, and empty probes were removed based on the annotation information contained in each platform file. When multiple probes matched the same gene, the average expression value was used to determine the gene’s expression level. To eliminate or minimize technical variability, the original expression matrix was normalized using the function “normalizeBetweenArrays”, whose default method is "quantile". After the pre-processing steps, the next major analysis stage is to identify differentially expressed genes. The “limma” package is an R package for analysing designed experiments and the assessment of differential expression. The lmFit (method = "ls") functions in the limma package was used to screen differentially expressed miRNAs (DEMis) and mRNAs (DEMs) in periodontitis cases and healthy controls. For purpose of reducing the false positive rate, the *p* value was adjusted by utilizing Benjamini–Hochberg false discovery rate (FDR) method [13]. The R script we used to pre-process and assess differential expression is detailed in Additional file 1: Data S1. The DEMs were screened for those with adjusted *p* value < 0.05 and |log FC| ≥ 0.75, and the DEMis were screened for those with adjusted *p* value < 0.05 and |log FC| ≥ 1. These were visualized using volcano maps by the plot in R.

miRNet 2.0, which is an online tool based on three bioinformatic algorithms (miRTarBase v8.0, TarBase v8.0, miRecords), was utilized to predict the potential target genes of aberrant miRNAs. A combined analysis of DEMs and target genes of DEMis was conducted by drawing Venn diagrams. Overlapping genes were considered significant DEMs in the regulatory network.

### Gene Ontology and Kyoto Encyclopedia of Genes and Genomes pathway enrichment analysis

The Gene Ontology (GO) database is the world’s largest source of information on the functions of genes [14]. The Kyoto Encyclopedia of Genes and Genomes (KEGG) is a database resource for understanding the high-level functions and utilities of biological systems from molecular-level information [15–17]. GO enrichment analysis and KEGG pathway analysis of significant DEMs were performed using the “enrichplot” and “ggplot2” packages in R. Statistical significance was set at adjusted *p* value < 0.05.

### Protein–protein interaction network construction and analysis

To gain insight into the interactions of the proteins, a protein–protein interaction (PPI) network was constructed. The DEMs were uploaded to the Search Tool for the Retrieval of Interacting Genes (STRING, https://string-db.org/), a database covering 9,643,763 proteins from 2,031 organisms. The results with a score of > 0.4 were imported into Cytoscape v.3.8.2 [18, 19]. The CytoHubba plugin was then used to identify the top 10% DEMs ranked by the topological analysis method of degree connectivity, and the Molecular Complex Detection (MCODE) plugin was used to filter out significant modules with a degree cut-off of 2, node score cut-off of 0.2, k-Core of 2, and maximum depth of 100.

### Construction of the miRNA–mRNA–TF regulatory network

Differential gene expression is achieved through complex regulatory networks, which are partly controlled by two types of transregulators: TFs and miRNAs. MiRNAs and TFs act as transregulators that control gene regulatory networks in a dependent or independent way [20, 21]. The plugin iRegulon in Cytoscape software, which identifies master regulators (transcription factors) and direct target genes in a human gene signature, was used to identify TFs in the miRNA–target regulatory network. In this study, potential TFs in relation to the significant DEMs were predicted with enrichment score threshold = 4.0, ROC threshold for AUC calculation = 0.05, rank threshold = 5000, minimum identity between orthologous genes = 0.05 and maximum FDR on motif similarity = 0.001. Subsequently, the miRNA–mRNA–TF network was constructed by loading all the DEMi-DEM pairs and TF-DEM pairs into Cytoscape software, which was used to visualize all the pairs at once.

## Results

### DEMis and DEMs screening in periodontitis

**Fig. 1:**
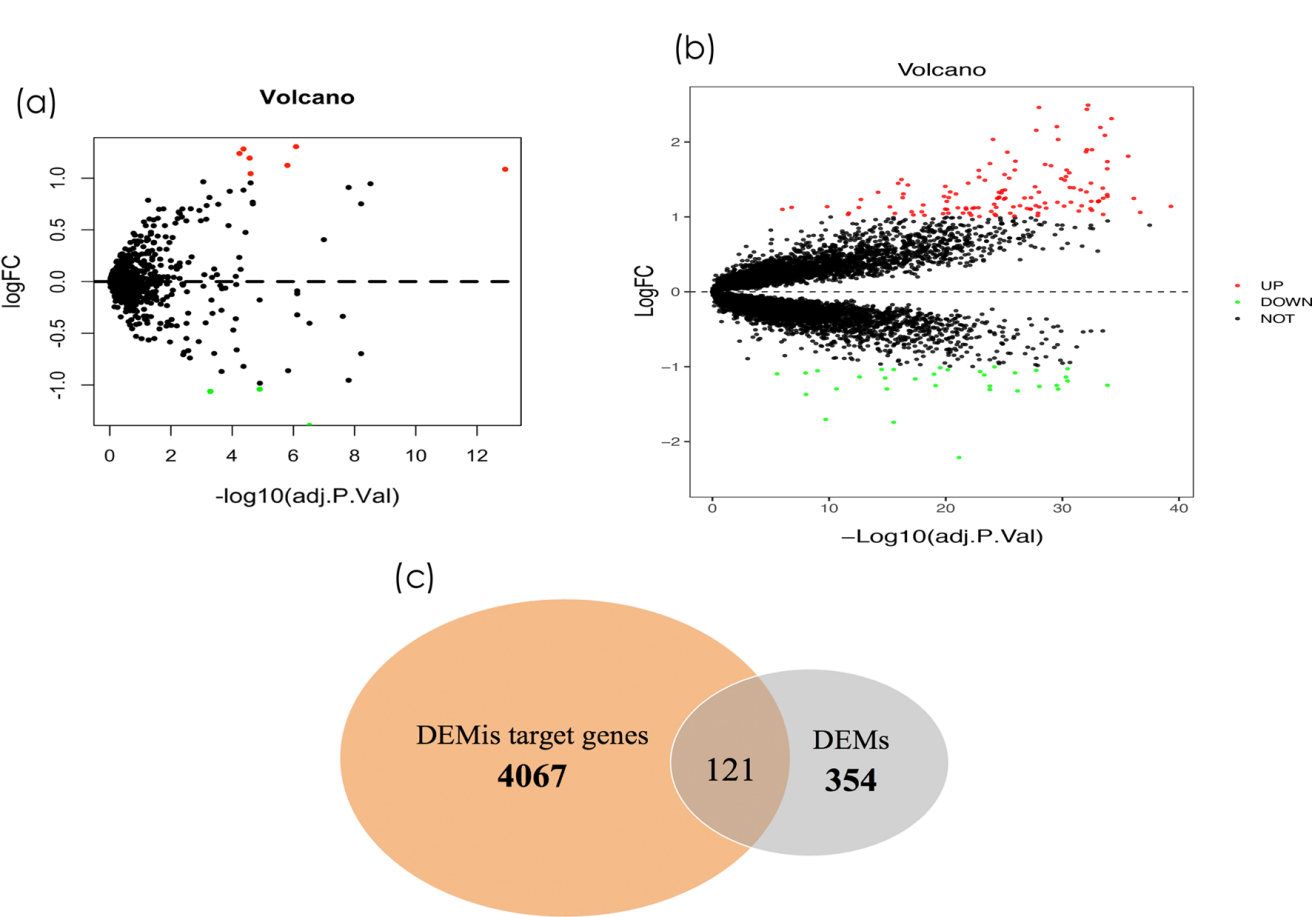
Identification of differentially expressed miRNAs (DEMis) and mRNAs (DEMs). **a**, **b** Volcano map of DEMis and DEMs. The x-axis indicates the log FC, and the y-axis indicates the log10 (adjusted *p* value). Red spots represent upregulated genes; green spots represent downregulated genes. **c** A Venn-diagram between DEMis target genes and DEMs

GSE54710 is a miRNA expression profile dataset that was downloaded from the NCBI GEO database. It contains 41 healthy gingival tissue samples and 159 tissue samples of periodontitis. Ten DEMis were obtained after the two groups of samples were screened based on the following criteria: adjusted *p* value < 0.05 and |log FC| ≥ 1. Four upregulated miRNAs and six downregulated miRNAs were included, and these DEMis were subsequently assessed. All ten DEMis are presented in Additional file 2: Table S1. A volcano map was drawn to show the distribution of differential miRNA expression between periodontitis and healthy controls (Fig. 1a).

We intended to identify the DEMs in periodontal and healthy samples using the NCBI GEO database. The GSE16134 dataset is a mRNA expression profile dataset containing 41 healthy gingival tissue samples and 159 periodontitis tissue samples. DEMs with adjusted *p* value < 0.05 and |log FC| ≥ 0.75 were identified. Consequently, 345 upregulated and 131 downregulated DEMs were identified. The top 20 upregulated and downregulated genes are listed in Additional file 2: Table S2. A volcano map was drawn to show the distribution of differential mRNA expression between periodontitis and healthy controls (Fig. 1b).

A total of 5632 target genes were identified from the miRNet database. A total of 121 genes were identified as the intersecting genes between these target genes and the DEMs (Fig. 1c). Therefore, a total of 121 DEMs, including 94 upregulated and 27 downregulated genes, were identified as the final sets of significant DEMs.

### GO and KEGG pathway enrichment analysis of DEMs

**Fig. 2:**
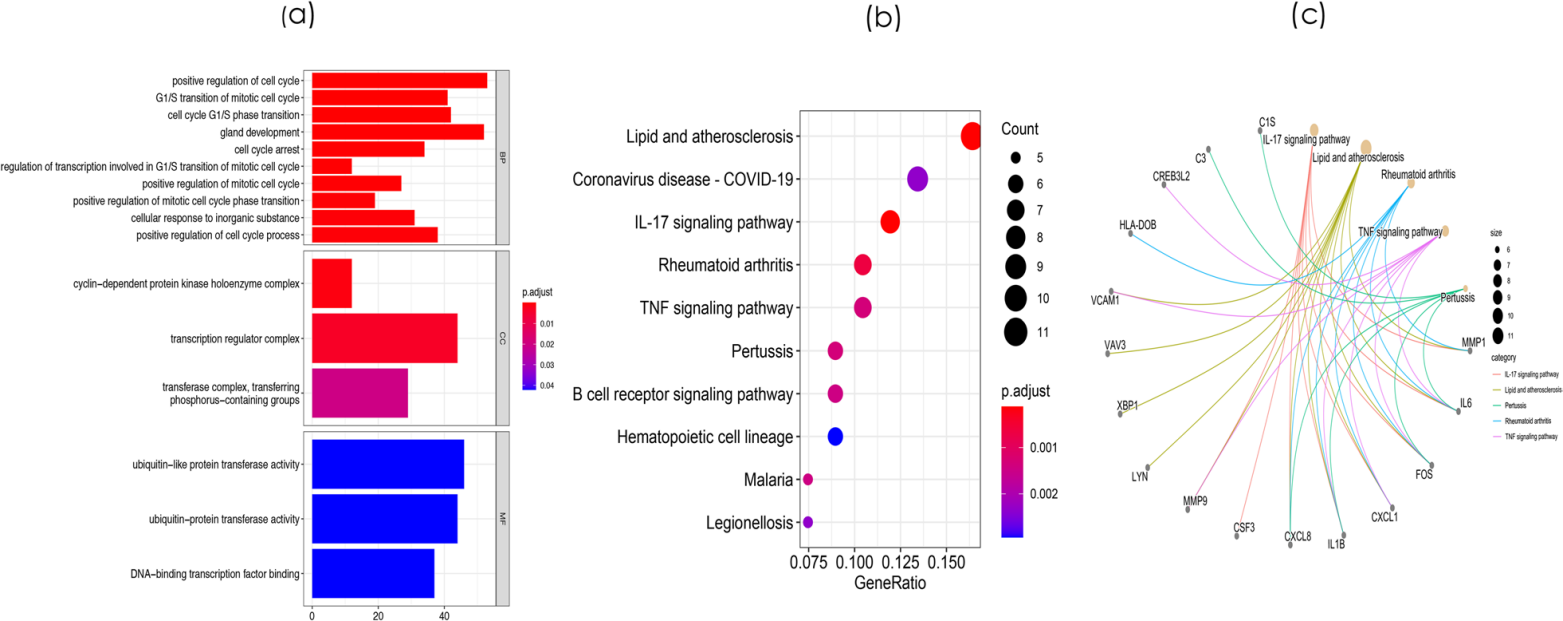
Functional enrichment analyses of differentially expressed genes (DEMs). **a** GO analysis of the DEMs. **b**. The KEGG pathway enrichment analysis of DEMs. **c** The relationship between enriched mRNAs in the KEGG pathway enrichment analysis

To further elucidate the function of mRNAs in the regulatory network, we used the “enrichplot” package in R to perform GO and KEGG analyses. GO analysis of the mRNAs in the regulatory network showed that the following BP terms were most significantly enriched in positive regulation of the cell cycle, gland development, and positive regulation of the cell cycle process. The most enriched MF terms included ubiquitin-like protein transferase activity, ubiquitin–protein transferase activity, and DNA–binding transcription factor binding. The most enriched CC terms included transcription regulator complex, transferase complex, transferring phosphorus-containing groups, and cyclin-dependent protein kinase holoenzyme complex (Fig. 2a and Additional file 3: Table S3). KEGG pathway analysis showed that mRNAs in the regulatory network were mainly involved in lipid and atherosclerosis, coronavirus disease-COVID-19, the IL-17 signaling pathway, and rheumatoid arthritis (Fig. 2b and Additional file 4: Table S4). The relationships between the mRNAs and the enriched KEGG pathways are shown in Fig. 2c.

### PPI network construction and analysis

**Fig. 3:**
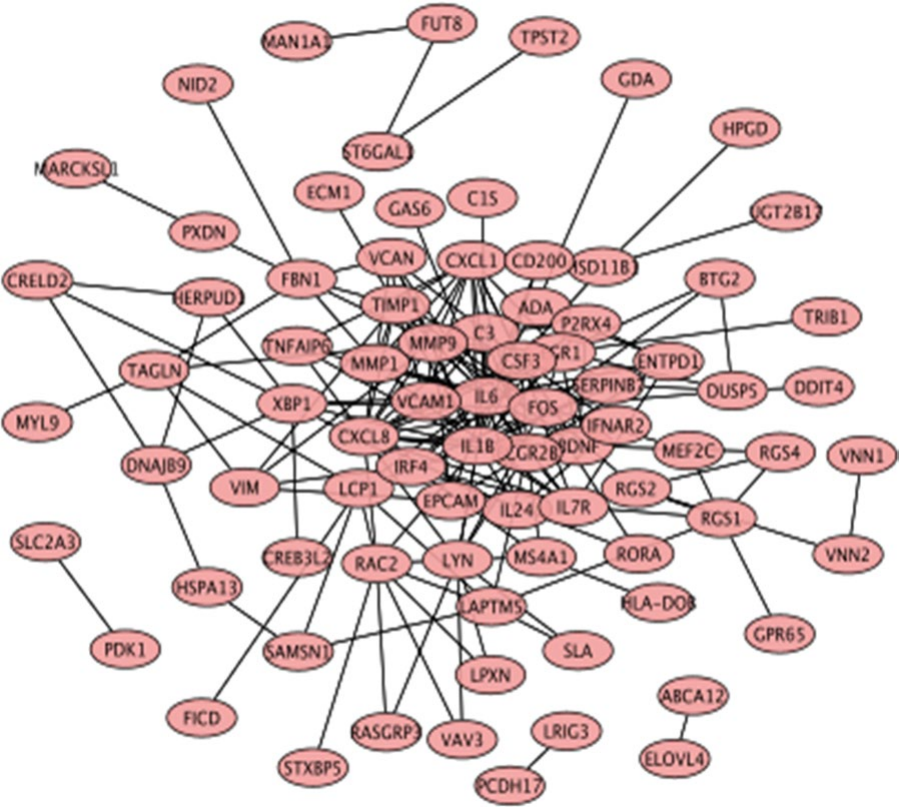
The protein–protein interaction (PPI) network of differentially expressed mRNAs (DEMs)

**Fig. 4:**
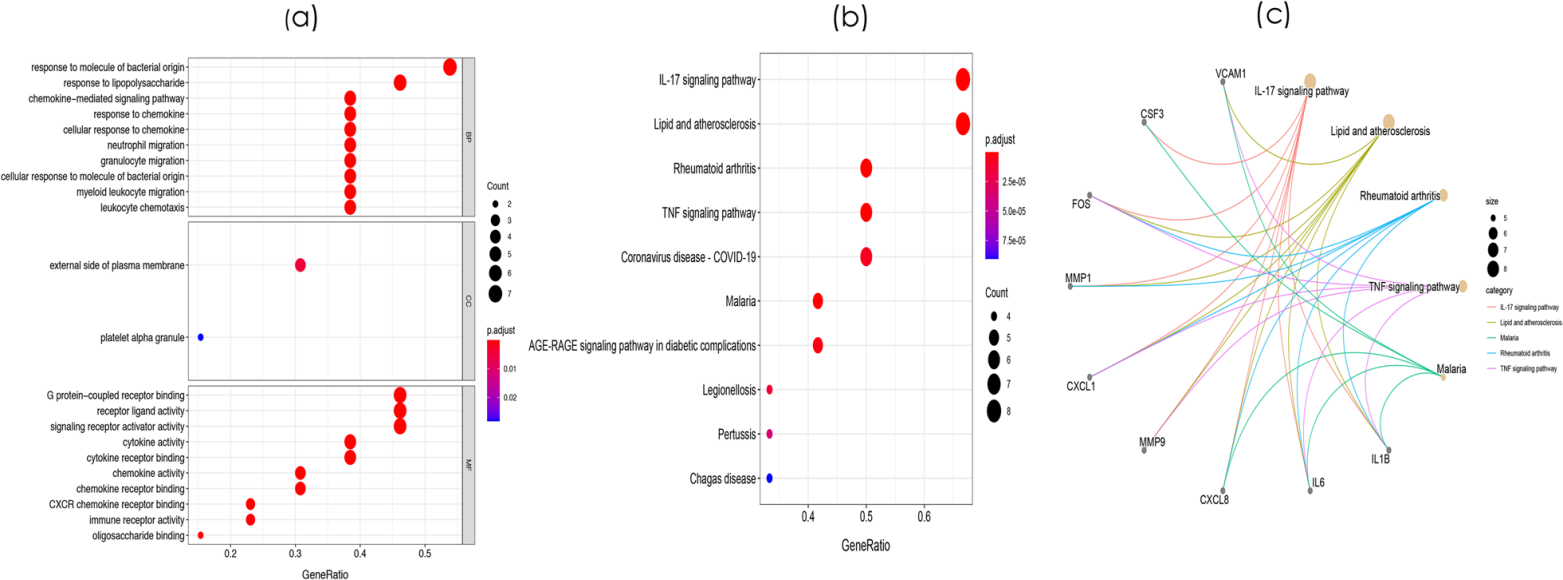
Functional enrichment analyses of the hub genes. **a** GO analysis of the hug genes. **b**. The KEGG pathway enrichment analysis of hug genes. **c** The relationship between enriched mRNAs in the KEGG pathway enrichment analysis

Subsequently, we mapped the DEMs identified based on the screening criteria into the STRING database and constructed a PPI network of these genes, with 81 nodes and 414 edges (Fig. 3). Hub proteins are those proteins in a network that are highly connected and are the master keys of regulation. To explore the hub genes in the PPI network, the node pairs were input into Cytoscape software and analysed using the CytoHubba plugin. The top 10% DEMs were *IL1B, IL6, CXCL8, MMP9, CXCL1, TIMP1, VCAM1, MMP1, FOS, CSF3, EGR1,* and *BDNF*. GO analysis results showed that hub genes were significantly enriched in G protein-coupled receptor binding and receptor ligand activity in the molecular function ontology, external side of plasma membrane and platelet alpha granule in cellular components ontology, and response to molecule of bacterial origin and response to lipopolysaccharide in biological processes ontology (Fig. 4a and Additional file 5: Table S5). KEGG analysis showed that hub genes were significantly enriched in the IL-17 signaling pathway, lipid and atherosclerosis, rheumatoid arthritis and TNF signaling pathway (Fig. 4b and Additional file 6: Table S6). The relationships between the mRNAs and the enriched KEGG pathways are shown in Fig. 4c. These pathways play an important role in the inflammatory response.

**Fig. 5:**
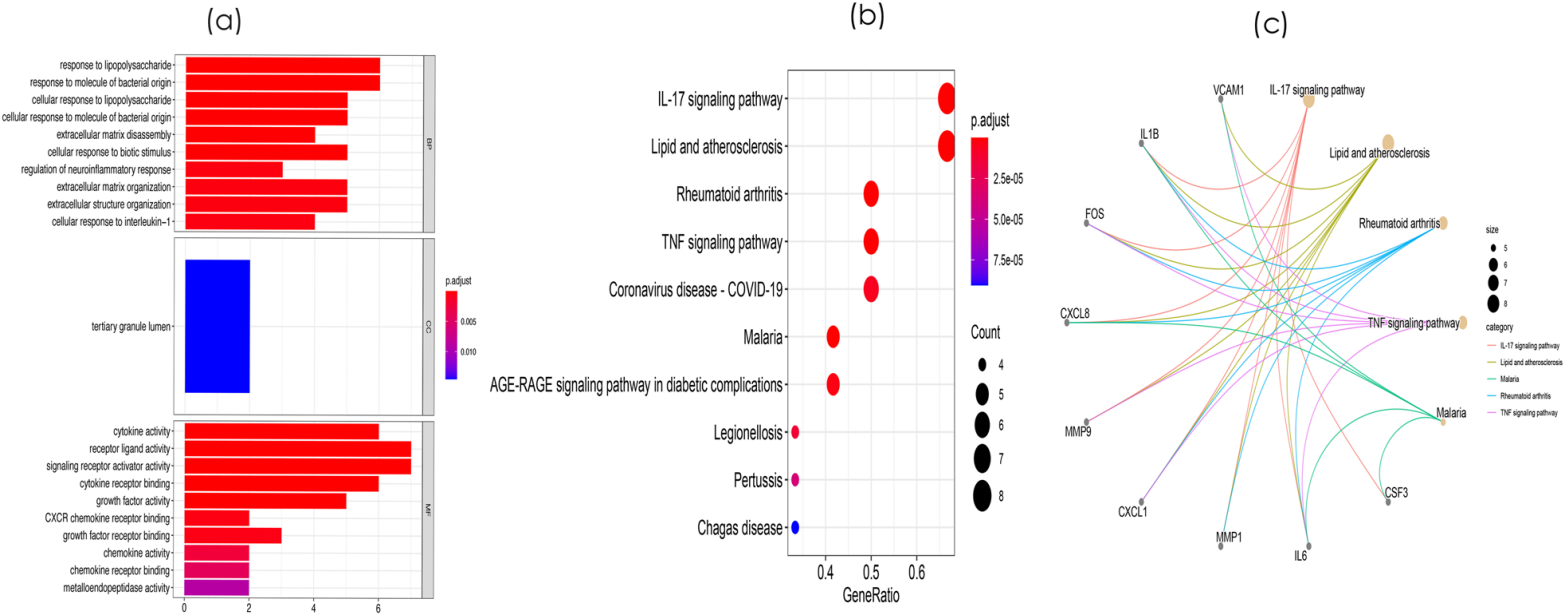
Functional enrichment analyses of Module 1. **a** GO analysis of the genes in Module 1. **b** The KEGG pathway enrichment analysis of the genes in Module 1. **c** The relationship between enriched mRNAs in the KEGG pathway enrichment analysis

MCODE was used to process the data downloaded from STRING to further mine gene clusters, and 4 cluster modules were obtained. The first gene cluster with the highest scores contained 12 nodes and 110 edges. GO analysis showed that these genes were mainly associated with response to lipopolysaccharide and response to molecule of bacterial origin in biological processes ontology; tertiary granule lumen in cellular components ontology; and receptor ligand activity and signaling receptor activator activity in the molecular function ontology (Fig. 5a and Additional file 7: Table S7). KEGG enrichment analysis revealed that the genes in this gene cluster were mainly involved in the IL-17 signaling pathway, lipid and atherosclerosis, rheumatoid arthritis, and TNF signaling pathway (Fig. 5b and Additional file 8: Table S8). The relationships between the mRNAs and the enriched KEGG pathways are shown in Fig. 5c.

### Construction of the miRNA–mRNA–TF regulatory network

**Fig. 6:**
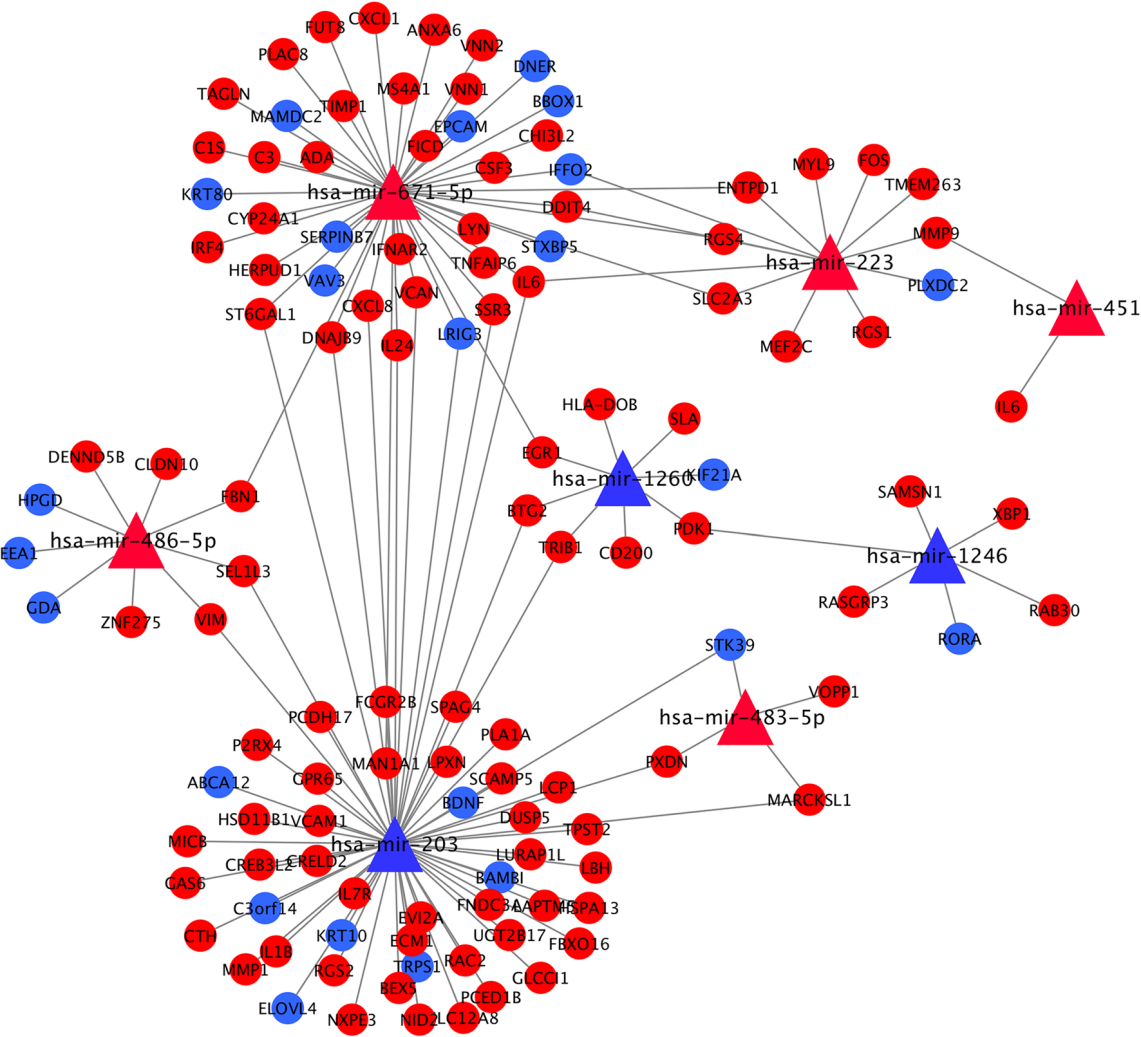
MiRNA–mRNA regulatory network. The triangles represent miRNAs, red circles represent mRNAs, and the upregulated nodes were exhibited by the red color, while the blue color exhibited the downregulated nodes

To further understand the regulatory relationship between DEMs and DEMis, a miRNA-mRNA regulatory network of 121 mRNAs and 8 miRNAs was constructed (Fig. 6). Understanding this interaction would help clarify the role of miRNAs in periodontitis. The following ten nodes with the highest degree were identified using the cytoHubba plugin in Cytoscape: hsa-miR-203, hsa-miR-671-5p, *IL6, ST6GAL1, VCAN, DNAJB9, MAN1A1, LRIG3, SSR3, and CXCL8*. These nodes were screened for a negative regulatory relationship between the miRNA and mRNA. In a negative regulatory relationship, upregulated target genes for DEMis are downregulated, or downregulated target genes for DEMis are upregulated. Consequently, hsa-miR-203/IL6 and hsa-miR-671-5p/*LRIG3* were identified as potential regulatory pathways in periodontitis.

**Fig. 7:**
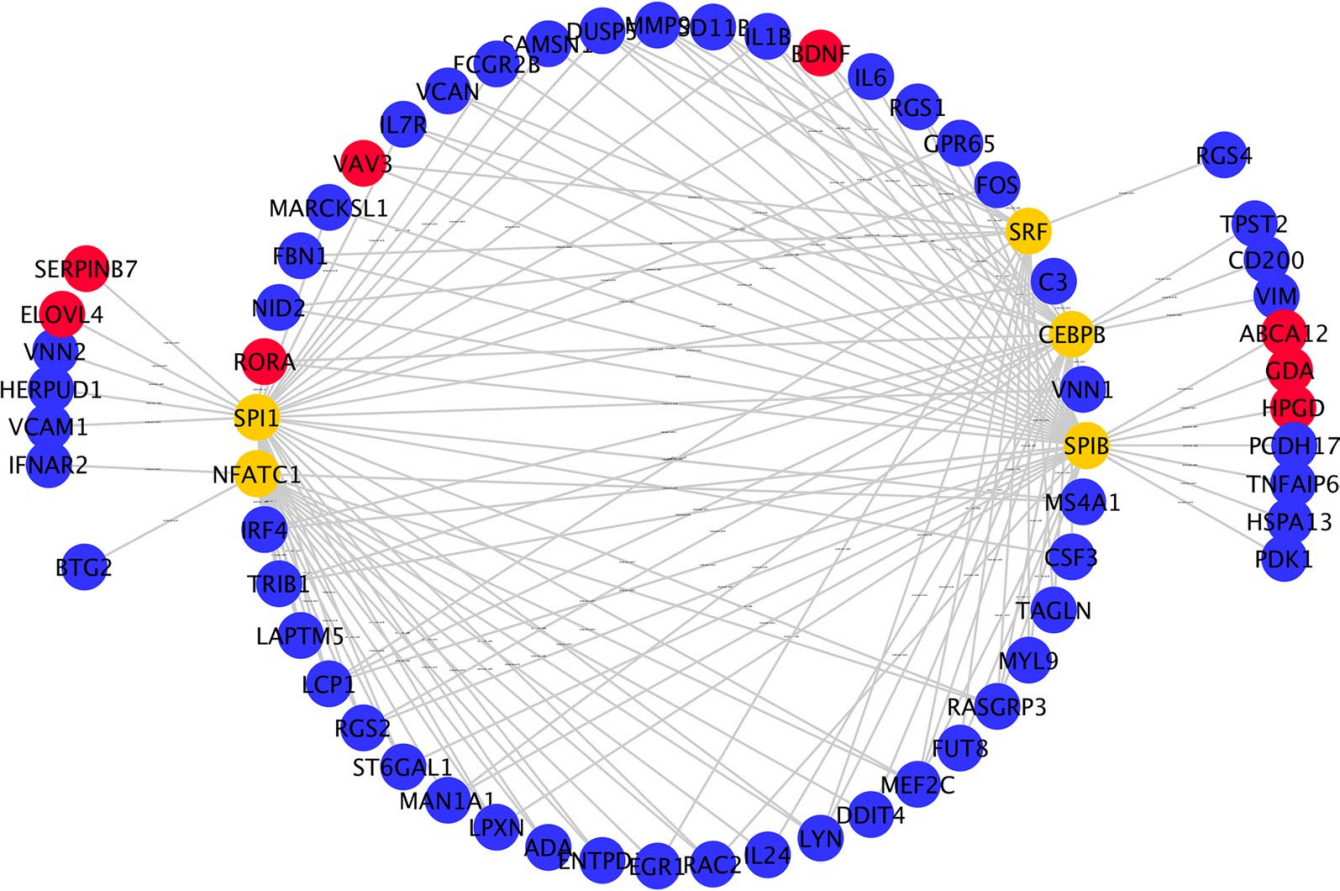
The TF–target regulatory network of differentially expressed mRNAs (DEMs). Yellow circles represent TFs, red circles represent upregulated mRNAs, and blue circles represent downregulated mRNAs

Five TFs were predicted to regulate DEMs by iRegulon analysis, including *SPI1, SPIB, CEBPB, NFATC1*, and *SRF.* To further elucidate these key mRNA functions, DEM-TF regulatory networks of 65 nodes and 131 interaction pairs were constructed (Fig. 7). *SPI1* targeted 41 genes, *SPIB* targeted 45 genes, *SRF* targeted 27 genes, *CEBPB* targeted 34 genes, and *NFATC1* targeted 46 genes. The top 10 nodes with the highest degree are listed in Table 1, among which RASGRP3, MEF2C, and LCP1 were jointly regulated by five TFs.

**Table 1:** Top 10 nodes with higher degree in transcription factor–mRNA regulatory network

Node name	Degree	Regulation	TF
RASGRP3	5	Up	SPI1, SPIB, SRF, CEBPB, NFATC1
MEF2C	5	Up	SPI1, SPIB, SRF, CEBPB, NFATC1
LCP1	5	Up	SPI1, SPIB, SRF, CEBPB, NFATC1
RGS2	4	Up	SPI1, SPIB, CEBPB, NFATC1
TRIB1	4	Up	SPI1, SPIB, CEBPB, NFATC1
IRF4	4	Up	SPI1, SPIB, CEBPB, NFATC1
LYN	4	Up	SPI1, SRF, CEBPB, NFATC1
DUSP5	4	Up	SPI1, SPIB, SRF, CEBPB
MMP9	4	Up	SPI1, SPIB, SRF, CEBPB
HSD11B1	3	Up	SPIB, SRF, CEBPB

**Fig. 8:**
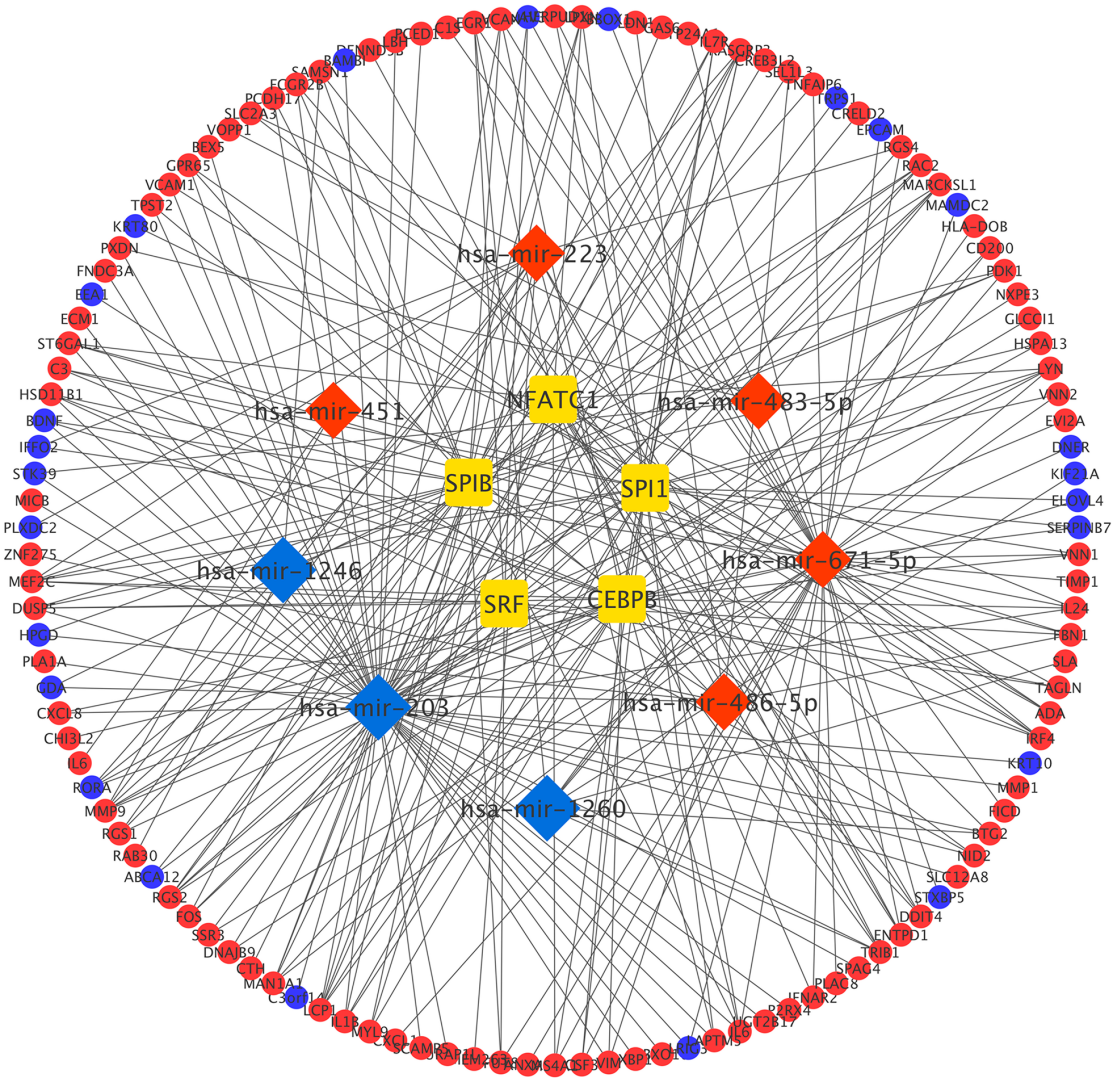
MiRNA–mRNA–TF regulatory network of differentially expressed mRNAs (DEMs). The diamonds represent miRNAs, the squares represent TFs, the circles represent mRNAs, and the upregulated nodes were exhibited by the red color, while the blue color exhibited the downregulated nodes

Finally, the miRNA–mRNA–TF regulatory network was established with 8 miRNAs, 121 DEmRNAs, and 5 TFs through Cytoscape 3.7.1 (Fig. 8).

## Discussion

Periodontitis is a complex infectious disease with various causes and contributing factors [22]. An increasing number of studies are now being conducted on the diagnosis and treatment of periodontitis. However, due to the limited understanding of the pathogenesis of periodontitis, the prognosis of patients with periodontitis remains poor. Recently, microarray technology has been used to reveal thousands of gene changes in the development of various diseases. Previous studies have examined the expression profile of periodontitis to identify DEMis and DEMs [23, 24]. Due to the limitations of the comparative analysis in independent studies, there are still problems with the interaction between DEMis and DEMs involved in periodontitis. In addition, in the context of genetic regulatory networks, the synergistic effects of transcription factors and miRNAs are largely unknown. To the best of our knowledge, this is the first attempt to integrate miRNA and mRNA expression profile data and construct a miRNA–mRNA–TF regulatory network. The identification and analysis of periodontitis-related genes, miRNAs and TFs may reveal the potential pathogenesis of periodontitis at the molecular level, which is helpful for identifying potential diagnostic and therapeutic strategies for future studies.

In our study, an integrative analysis of microarray and RNA-sequencing datasets was conducted, and a total of 121 DEMs were identified as significant elements for periodontitis. For the purpose of fully understanding the function and mechanism of these DEMs, we conducted GO and KEGG enrichment analyses utilizing the “enrichplot” packages in R. Gene Ontology analysis of DEMs showed that DEMs are mainly involved in positive regulation of the cell cycle, gland development, and positive regulation of the cell cycle process, and these biological processes mainly contribute to cardiac cell proliferation and differentiation. KEGG pathway analysis demonstrated that abnormal molecular expression of several pathways may contribute to the pathogenesis of periodontitis, such as the IL-17 signaling pathway. In previous studies, the activation of the IL-17 signalling pathway has been shown to be associated with periodontitis. A study performed by Satoru showed that IL-17A may promote the progression of periodontitis through proinflammatory cytokine production [25]. Moreover, IL-17 is essential for the maintenance of bone mass, as it orchestrates osteoclast differentiation and activation [26]. Kukolj et al. revealed that IL-17 inhibited both the proliferation and migration of periodontal ligament mesenchymal stem cells and decreased their osteogenic differentiation by activating ERK1/2 and JNK mitogen-activated protein kinases [27]. Therefore, inhibition of the IL-17 signalling pathway may be a therapeutic strategy for periodontitis.

Among the five TFs (*SPI1, SPIB, CEBPB, NFATC1*, and *SRF*) predicted in the present study. All these TFs have been verified as key modulators and potential therapeutic targets in a wide variety of inflammatory diseases. SPI-1 genes are responsible for the invasion of host cells, regulation of the host immune response, e.g., the host inflammatory response, immune cell recruitment and apoptosis, and biofilm formation [28]. SRIB has been previously reported to display anti-inflammatory functions by producing interleukin IL-9, and higher levels of IL-9 and SRIB were detected in gingivitis patients than in healthy individuals [29]. CEBPB is a member of the family of transcription factors that play roles in a wide range of cellular processes, such as cellular apoptosis, proliferation, adipocyte differentiation, carbohydrate metabolism and inflammation [30, 31]. Overexpression of CEBPB markedly increased the expression of the proinflammatory cytokines IL-6 and IL-8 in human periodontal ligament cells in response to LPS [32]. Li et al. demonstrated that SRF appears to be involved in the regulation of IL1B and CXCL8 [33].

Hsa-mir-203 and hsa-mir-671-5p were identified as hub miRNAs based on integrated network analysis. MiR-203 was first identified in the pathogenesis of psoriasis and is involved in various physiological and pathological processes [34]. Zhang et al. demonstrated that miR-203 overexpression suppresses *TRAF6*-induced IL-β, IL-6, and TNF-α activation in human renal mesangial cells and proximal tubular cell line cells [35]. A recent study showed that miR-203 protects against microglia-mediated brain injury by targeting the MyD88 protein to modulate the inflammatory response [36]. Furthermore, Wang et al. showed that miR-203 inhibits inflammation to alleviate myocardial ischaemia–reperfusion injury [37]. It has been reported that miR-671-5p represses cell proliferation, migration, invasion and the inflammatory response [38]. MiR-671 mimics ameliorated IL-1β-induced proliferation inhibition and apoptosis stimulation and alleviated the progression of osteoarthritis in mice [39]. However, whether miR-671-5p and miR-203 are implicated in the pathogenesis of periodontitis can be investigated in future studies.

Although we constructed a potential miRNA–mRNA–TF regulatory network by integrating multiple microarray datasets for the first time, our study is limited by the fact that we performed an integrated analysis based on only one microarray dataset and one RNA-seq dataset, which may reduce the credibility of miRNA–mRNA–TF coregulatory network analysis. In addition, the clinical significance of the predictions has not been evaluated by the receiver operating curve (ROC) using the area under the curve (AUC), and further work needs to be done in the future. Moreover, predictions were made based on public databases; further experiments are lacking to validate the analytical results as a firm basis.

## Conclusions

In this study, we performed an integrated analysis based on public databases to identify specific TFs, miRNAs, and mRNAs that may play a pivotal role in periodontitis. On this basis, a miRNA–mRNA–TF network was established to provide a comprehensive perspective of regulatory mechanism networks of periodontitis.

## Supplementary Information

**Additional file 1.** R scripts for preprocessing and evaluating differential expression.

**Additional file 2.** Key DEMis accessed from GSE54710 and GSE16134.

**Additional file 3.** GO analysis of the DEMs.

**Additional file 4.** The KEGG pathway enrichment analysis of DEMs.

**Additional file 5.** GO analysis of the hug genes.

**Additional file 6.** The KEGG pathway enrichment analysis of hug genes.

**Additional file 7.** GO analysis of the genes in Module 1.

**Additional file 8.** The KEGG pathway enrichment analysis of the genes in Module 1.

## Data Availability

The datasets (GSE54710 and GSE16134) generated and analysed during the current study are available in the GEO dataset repository. https://www.ncbi.nlm.nih.gov/gds.

## Abbreviations

BP: Biological process
CC: Cellular component
DEG: Differentially expressed genes
DEMis: Differentially expressed miRNAs
DEMS: Differentially expressed mRNAs
GEO: Gene Expression Omnibus
GO: Gene Ontology
KEGG: Kyoto Encyclopaedia of Genes and Genomes
LPS: Lipopolysaccharides
MCODE: Molecular Complex Detection
MF: Molecular function
MiRNA: MicroRNA
NCBI: National Center for Biotechnology Information
PPI: Protein–protein interaction
STRING: Search Tool for the Retrieval of Interacting Genes
TF: Transcription factors
