# Supplementary material for: Identification of microRNA–mRNA–TF regulatory networks in periodontitis by bioinformatics analysis
Source: BMC Oral Health. 2022 Apr 9;22:118. doi: 10.1186/s12903-022-02150-0 (PMC8994180; doi:10.1186/s12903-022-02150-0)
Supplement: Supplementary file 2 — Additional file 2. Key DEMis accessed from GSE54710 and GSE16134. [file 12903_2022_2150_MOESM2_ESM.docx]

**Table S1. Key DEMis accessed from GSE54710**

| miRNA_ID | logFC | P Value | Adjusted P value |
| --- | --- | --- | --- |
| hsa-miR-3917 | 1.085309938 | <0.001 | <0.001 |
| hsa-miR-1246 | -1.390929316 | <0.001 | <0.001 |
| hsa-miR-486-5p | 1.30391312 | <0.001 | <0.001 |
| hsa-miR-1260 | -1.04054174 | <0.001 | <0.001 |
| hsa-miR-483-5p | 1.043113657 | <0.001 | <0.001 |
| hsa-miR-671-5p | 1.193440192 | <0.001 | <0.001 |
| hsa-miR-451 | 1.281609232 | <0.001 | <0.001 |
| hsa-miR-223 | 1.238010714 | <0.001 | <0.001 |
| hsa-miR-203 | -1.061388711 | <0.001 | <0.001 |
| hcmv-miR-UL70-3p | 1.123636686 | <0.001 | <0.001 |

**Table S2. Top 40 differentially expressed mRNAs, half upregulated, half downregulated.**

| Gene_ID | logFC | P Value | Adjusted P value | Gene_ID | logFC | P Value | Adjusted P value |
| --- | --- | --- | --- | --- | --- | --- | --- |
| Top 20 downregulated mRNAs | | | | Top 20 upregulated mRNAs | | | |
| DSC1 | -2.212 | <0.001 | <0.001 | MZB1 | 2.492 | <0.001 | <0.001 |
| FLG2 | -1.744 | <0.001 | <0.001 | TNFRSF17 | 2.461 | <0.001 | <0.001 |
| KRT2 | -1.704 | <0.001 | <0.001 | IGLL5 | 2.437 | <0.001 | <0.001 |
| LOR | -1.371 | <0.001 | <0.001 | IGK | 2.312 | <0.001 | <0.001 |
| BPIFC | -1.324 | <0.001 | <0.001 | SPAG4 | 2.204 | <0.001 | <0.001 |
| ELOVL4 | -1.305 | <0.001 | <0.001 | IGHM | 2.194 | <0.001 | <0.001 |
| CLDN20 | -1.299 | <0.001 | <0.001 | LOC100293211 | 2.156 | <0.001 | <0.001 |
| CALML5 | -1.298 | <0.001 | <0.001 | FAM46C | 2.088 | <0.001 | <0.001 |
| FLG | -1.297 | <0.001 | <0.001 | CXCL6 | 2.034 | <0.001 | <0.001 |
| NEFL | -1.265 | <0.001 | <0.001 | CD79A | 2.033 | <0.001 | <0.001 |
| AADAC | -1.260 | <0.001 | <0.001 | CXCL1 | 1.897 | <0.001 | <0.001 |
| SLC27A6 | -1.253 | <0.001 | <0.001 | IGLV1-44 | 1.896 | <0.001 | <0.001 |
| RORA | -1.248 | <0.001 | <0.001 | CD27 | 1.869 | <0.001 | <0.001 |
| ATP6V1C2 | -1.191 | <0.001 | <0.001 | SLAMF7 | 1.864 | <0.001 | <0.001 |
| ABCA12 | -1.164 | <0.001 | <0.001 | CXCR4 | 1.809 | <0.001 | <0.001 |
| LCE2B | -1.151 | <0.001 | <0.001 | LAX1 | 1.744 | <0.001 | <0.001 |
| NPR3 | -1.139 | <0.001 | <0.001 | CHST2 | 1.738 | <0.001 | <0.001 |
| MAMDC2 | -1.137 | <0.001 | <0.001 | LOC101929272 | 1.712 | <0.001 | <0.001 |
| SLC16A9 | -1.111 | <0.001 | <0.001 | CYTIP | 1.700 | <0.001 | <0.001 |
| EPCAM | -1.100 | <0.001 | <0.001 | CPNE5 | 1.683 | <0.001 | <0.001 |
